# Tumor in the Lumbar Region

**Published:** 1862-12

**Authors:** J. F. Norton

**Affiliations:** Fort Edward


ART. III.—*Tumor in the Lumbar Region.* By J. F. Norton, M. D.

Fort Edward, October 20th, 1862.

I submit the following case, believing it of sufficient interest to communicate, from the fact that I have never before seen a case, nor do I now recollect of having read of a case in any of our journals.

Some time since I was called to see an Irish laboring man, who complained that he was losing the use of his lower limbs. He referred the cause of his difficulty to an injury of the back, caused from lifting a heavy weight, "feeling something give way at the time," as he expressed it; he was able however to walk home from the iron furnace where he labored. He contented himself by using liniments and such like treatment until I saw him, some ten days after the injury. I directed him to get upon his feet and walk, which he did with some difficulty, and the assistance of a cane. He assumed a stooping position, and his walk was tottering, with a twitching of the legs. He complained that his legs were cold and numb; pain in the back and head, with deranged vision, while in an erect position; while recumbent, he remained comparatively easy. Upon examination I found a soft, puffy tumor, or rather fullness, upon the left side of the spinal column, and between the last dorsal and first lumbar vertebræ; the tumor was not tender to the touch, neither was the skin discolored. I placed him in a prone position, and upon passing my hand upon the tumor, was surprised to see it disappear, but not without an aggravation of the symptoms complained of while standing, there was severe nausea, with a tendency to cramping of the legs while continuing the pressure. There evidently appeared to be a space between the vertebræ at this point, in which I could place the end of my finger. The tumor readily re-appeared upon assuming an erect posture. I placed him upon tonics, with the arsenicalis liquor, and then directed my attention to confining the tumor, after reduction; this was a somewhat difficult task, and I had not fully succeeded when he was obliged to be removed to the County-House, thus losing sight of him before I could mature my plan of treatment; this, however, the attending physician promised to do, and report accordingly. I intend to visit the case soon, and will communicate the result.

P. S.—I should be grateful to know your opinion, and any remark you may think proper to make, if you have had experience in such cases.

A tumor receding upon taking the horizontal position and applying pressure, which would again re-appear when erect, and attended by the other circumstances and symptoms described, would make us mistrust a hernia. Its very unusual location, if of this character, would at once reflect some doubt, and the most careful examination only, could render the diagnosis satisfactory. Ruptures have been met with in almost all situations in the walls of the abdomen; and Colquet, describes a case occurring in the lumbar region. These different protrusions at various unusual locations are met with, as the result of injuries, and could hardly be believed, to take place without arising from direct traumatic cause; the lifting, might possibly have been adequate for its production. If the tumor is of this nature, it is of great interest, and we hope that its character will not be left in doubt.—Ed.

